# Obesity and Preference-Weighted Quality of Life of Ethnically Diverse Middle School Children: The HEALTHY Study

**DOI:** 10.1155/2013/206074

**Published:** 2013-06-19

**Authors:** R. P. Treviño, T. H. Pham, S. L. Edelstein

**Affiliations:** ^1^Social and Health Research Center, 1302 South Saint Mary's Street, San Antonio, TX 78210, USA; ^2^Biostatistics Center, The George Washington University, 6110 Executive Boulevard, Suite 750, Rockville, MD 20852, USA

## Abstract

To date, studies examining the relation between body mass index percentile (BMI%) categories and health-related quality of life (QOL) measurements have not reported preference-weighted scores among ethnically diverse children. We report the associations between BMI% categories and preference-weighted scores among a large cohort of ethnically diverse sixth grade children who participated in the HEALTHY school-based type 2 diabetes risk factor prevention study. Health Utility Index 2 (HUI2) and Health Utility Index 3 (HUI3) and the feeling thermometer (FT) were the preference-weighted QOL instruments used to measure student's preference scores. Of 6358 consented students, 4979 (78.3%) had complete QOL, height, weight, and covariate data. Mean (SD) preference scores were 0.846 (0.160), 0.796 (0.237), and 0.806 (0.161) for the HUI2, HUI3, and FT, respectively. After adjusting for age, sex, blood glucose and insulin, Tanner stage, race/ethnicity, family history of diabetes, and educational attainment, children with severe obesity (>99%) had significantly lower preference scores compared to normal weight on all three instruments (HUI2 *P* = 0.013; HUI3 *P* = 0.025; and FT *P* < 0.001). Obese and severe obese categories were significantly associated with lower HUI2 functional ratings in the mobility domain and with lower HUI3 functional ratings in the speech domain.

## 1. Introduction

The growing literature on the effects of obesity on children's self-reported health-related quality of life (HRQOL) has shown negative associations between some body mass index percentile (BMI%) categories and HRQOL [1–9]. These studies, however, have mainly been clinic based, used small samples at the extreme ends of the BMI distribution, and included limited numbers of minority children, who suffer the greatest burden from obesity [10]. Although there were two community-based studies that analyzed the relationship between BMI and HRQOL among ethnically diverse children, the percentages of African American and Hispanic children were small and the HRQOL instrument used were health status and not preference weighted [3, 6]. 

Preference-weighted quality of life (QOL) measurements, also known as quality-adjusted life-years (QALYs), is the measurement recommended by the US Panel on Cost-Effectiveness in Health and Medicine for cost-effectiveness analysis (CEA) [11]. QALY measures are based on economic theories (utility and game theories) that quantify the way in which people make choices when faced with uncertainty [12]. Health status instruments ask people to describe the level of disability in several domains (e.g., vision, hearing, and mobility). QALY measures provide additional information, asking people to determine the risk of death they are willing to take to improve that level of disability. QALY combines length and quality of life into a single measure of health outcome. QALY scores usually range from 0 to 1, where 0 represents death and 1 represents perfect health. For example a score of  0.80 means that an individual is willing to give up  0.20 of their life to live in perfect health. There are states worse than death, which give negative preference-weighted scores [13]. The QALY classification system intention is to put a worth or “monetary term” to health outcomes. By measuring cost and health outcomes, economists can determine how much health an investment buys. 

To date, studies examining the relation between BMI% categories and HRQOL have not reported preference-weighted scores among ethnically diverse children. We report the associations between BMI% categories and preference-weighted scores among a large cohort of ethnically diverse sixth grade children who participated in the HEALTHY school-based type 2 diabetes risk factor prevention study [14]. Health Utility Index 2 (HUI2) and Health Utility Index 3 (HUI3) and the feeling thermometer (FT) were the preference-weighted QOL instruments used to measure student's preference scores. We hypothesized that BMI% categories are negatively associated with preference-weighted QOL scores in ethnically diverse middle school children.

## 2. Methods

### 2.1. The Trial

The HEALTHY intervention, which focused on environmental and individual changes in nutrition, physical activity, and behavior, was conducted in 42 middle schools recruited by 7 field centers across the USA. The goal of HEALTHY was to reduce or moderate 4 risk factors for type 2 diabetes: BMI%, waist circumference, and fasting blood glucose and insulin levels. HEALTHY was initiated among sixth grade students at the beginning of the 2006-2007 school year and continued through the eighth grade in the 2008-2009 school year. Data collected during the 2006-2007 school year was used for this analysis. Detailed methods and primary results of the HEALTHY trial have been published elsewhere [14]. 

### 2.2. Participants

 Eligible students were in the sixth grade of the 42 middle schools. Eligible schools had at least 50% minority students, defined as African American, Hispanic, or American Indian, or at least 50% of the students eligible for free or reduced price meals from the National School Lunch Program (NSLP). 

### 2.3. Measures

HEALTHY was approved by the Institutional Review Boards at all the seven study sites. Federalwide Assurance to conduct federally funded research was obtained for all schools in the study. Written parent/guardian consent and student assent were obtained for all participants. 

Measures were collected at baseline from sixth grade students during the 2006-2007 school year. The HUI2 [15], HUI3 [16], and the FT from the EuroQOL [17] were the preference-weighted QOL instruments used. The HUI instrument asks respondents to rate their current level of health function across a number of domains. The HUI2 assesses seven health domains: sensation, mobility, emotion, cognition, self-care, pain, and fertility. The fertility domain questions are optional and were not used in this study [13]. The HUI3 assesses 8 domains: vision, hearing, speech, ambulation, dexterity, emotion, cognition, and pain. Preference scores are assigned to these ratings by use of utility scoring rules that have been developed by use of samples from the general public [15, 16]. The reading level of the questions used in HUI2 and HUI3 is grade six [18]. Reliability and validity of the instrument have been shown to be acceptable in children as young as 10 years [19]; several studies, proxy and self-reported, have used the instrument to assess preference-weighted scores among children younger than 11 [19–24]. 

The FT is another instrument that can be used to assess preference scores. We asked participants to rate how good or bad their current health is on a 0 to 100 scale, where 0 represented “worst imaginable health” and 100 represented “best imaginable health.” FT ratings were divided by 100 in order to make them comparable to HUI scores. The FT has been shown to be reliable and valid in children as young as 8 year [25, 26]; Civita et al. have reported that the FT has been used with children as young as 7 years of age [23]. 

The HUI questionnaire was administered to the students under staff supervision by use of “Personal Digital Assistants.” The FT was administered in a paper/pencil format. Both instruments were available in either English or Spanish.

 Weight and height were measured once without shoes by trained and certified HEALTHY staff. Weight was measured by use of SECA Alpha 882 digital scales (SECA Corporation, Chino, CA, USA); height was measured by use of PE-AIM-101 stadiometers (Perspective Enterprises, Portage, MI, USA). BMI% was calculated from the Centers for Disease Control and Prevention BMI-for-age-and-sex growth charts and categorized as underweight (<5), normal weight (5 to <85), overweight (85 to <95), obesity (95 to ≤99), and severe obesity (>99) [27]. 

Fasting blood was drawn to determine glucose and insulin levels. We categorized fasting glucose as <100 mg/dL, 100 to <110 mg/dL, 110 to <126 mg/dL, and 126+ mg/dL [28], and fasting insulin as <30 *μ*U/mL and 30+ *μ*U/mL [29]. We collected self-report information on student age, gender, pubertal status (by use of the Tanner scale), and race/ethnicity. Parents provided information about family history of diabetes and, as a measure of socioeconomic status, the highest educational grade attained in the household. Age, gender, race/ethnicity, and parental education have been commonly controlled for in the literature that has studied the relationship between children's self-reported HRQOL and BMI% [1–3, 5–8].

### 2.4. Analyses

Exclusion criteria for this analysis were the following: children who were underweight, age 13 years or older, and had missing QOL scores and covariate data. Children who were underweight (<5 BMI%) were excluded because of the small proportion, the mean QOL scores for underweight and normal weight were nearly similar, and the study aim was to evaluate the relationship between QOL scores and greater BMI% ranges. Because the average age of a sixth grade student is 11 years, students age 13 years or more may have been retained in the sixth grade for reasons other than health and thus might have influenced QOL scores independent of BMI% categories.

All statistical analyses were performed using SAS 9.2 (SAS Institute Inc., Cary, NC) by the George Washington University Biostatistics Center. We report means and proportions for descriptive statistics. Comparisons were performed using analysis of variance for self-ratings of preference-weighted QOL scores, and a *P* value <0.05 was considered significant with no adjustment for multiple comparisons.

We used linear mixed model analysis that accounts for the clustering of students within schools to assess the association between BMI% categories and QOL scores adjusted for covariates. Covariates included were age, sex, blood glucose and insulin, Tanner stage, race/ethnicity, family history of diabetes, and educational attainment. For covariates that had only 2 categories, a single difference in QOL score and *P* value was reported compared to the reference group. For covariates that had more than 2 categories, an overall *P* value as well as differences and *P* values for the named categories versus the reference group was reported. Reference groups were normal weight, fasting glucose <100 mg/dL, fasting insulin <30 *μ*U/mL, age of 11 years, female, Tanner stage 1, no family history of diabetes, non-Hispanic white children, and college graduate. 

## 3. Results

### 3.1. Participant Response Rate

Of the approximately 11,158 sixth grade students at 42 schools, 6358 (57.0%) had written parent/guardian consent and student assent prior to baseline measurement. Ninety-nine children were underweight (1.6%), 279 were 13 years or older (4.4%), 389 had missing preference-weighted QOL data (6.1%), and 743 had missing covariate data (11.7%). After applying the exclusion criteria, 4979 students comprised the analytic sample (44.6% of the 11,158 sixth grade students enrolled or 78.3% of the 6,358 students with consent/assent). Of the analytical sample, 92.2% answered the questionnaire in English and 7.8% in Spanish. Students who were excluded from the analysis were more likely to be male (22.8% versus 20.7%; *P* = 0.045). No differences were seen for BMI% categories (*P* = 0.13) or for the other variables we collected. 

### 3.2. Characteristics of Participants

Table 1 shows the characteristics of students. The rate of combined obesity and severe obesity was 30.5% (23.6% obesity and 6.9% severe obesity). African American and Hispanic children made up 78.8% of the participants, and 27.0% were from families with low educational attainment (as measured by no high school diploma). The average percent of students eligible for NSLP in the schools that participated in the HEALTHY study was 76.6%. 

### 3.3. Preference-Weighted QOL Scores

The mean (SD) preference-weighted QOL scores were 0.846 (0.160) for the HUI2, 0.796 (0.237) for the HUI3, and 0.806 (0.161) for the FT.  Table 2 shows the unadjusted scores stratified by clinical and demographic categories. BMI% categories were negatively associated with QOL scores on all three instruments (HUI2 *P* < 0.001, HUI3 *P* = 0.004, and FT *P* < 0.001). Scores for obese children (HUI2 *P* = 0.007, HUI3 *P* = 0.026, and FT *P* < 0.001) and severely obese (HUI2 *P* < 0.001, HUI3 *P* < 0.001, and FT *P* < 0.001) children were significantly lower than those for normal weight children. When overweight children were compared with normal weight, HUI2 and HUI3 scores showed no significant difference. Other clinical and demographic categories that showed significance after being stratified by QOL scores are shown in Table 2. 

Table 3 shows the adjusted associations between clinical and demographic categories and the 3 QOL score differences derived from the mixed model analyses. Only children with severe obesity remained with significantly lower QOL scores, compared to normal weight, on all three instruments (HUI2 *P* = 0.013; HUI3 *P* = 0.025; and FT *P* < 0.001). Obese and overweight children did not have significantly lower scores than normal weight children on the HUI2 and HUI3. FT showed significance among all BMI% categories. 

Hispanic and black children had significantly lower QOL scores than non-Hispanic white children on the HUI2 and HUI3 instruments but not on the FT. Other characteristics that were significantly associated with one or some of the QOL scores were age, gender, and Tanner stage. 

### 3.4. Domains Associated with Lower Preference-Weighted QOL Scores

**Figure 1:**
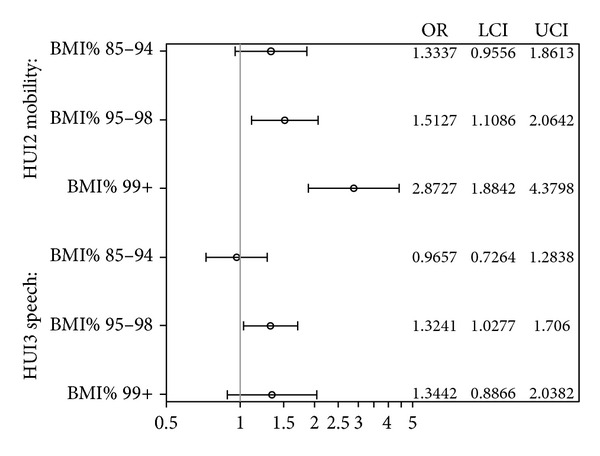
Odds ratios by level of Health Utility Index functioning for BMI category compared with normal weight, adjusted for gender, race/ethnicity, tanner stage, household education, and insulin.

BMI% categories were significantly associated with lower HUI2 functional ratings in the mobility domain and with lower HUI3 functional ratings in the speech domain (see Figure 1). The other domains showed no significance between BMI% categories and lowered functioning scores. Obese and severely obese children were 1.5 and 2.9 times more likely, respectively, to present lower levels of HUI2 mobility. Both obese and severely obese children were 1.3 times more likely to show lower levels of HUI3 speech, although findings were not statistically significant in severely obese children. 

## 4. Discussion

This is the first school-based study to measure preference-weighted QOL scores in a large, ethnically diverse population of sixth grade students. The purpose was to determine the association between preference-weighted scores using three instruments (HUI2, HUI3, and FT) and BMI% among mostly minority children. This is important because minority children suffer the greatest burden of obesity. Students who were severely obese rated their preference-weighted QOL in all three instruments significantly lower than those who were normal weight, before and after the adjustment for demographic factors and glucose and insulin levels. Scores based on the FT instrument were significantly lower among overweight, obese, and severely obese students than their normal weight counterparts.

A number of studies have found that children with combined obesity and severe obesity report significantly lower QOL scores than do normal weight children [1–8], but none to our knowledge have studied a range of BMI categories and preference-weighted QOL instruments among a large population of minority children. The current paper is the first to suggest that preference-weighted QOL function ratings decrease clinical at severe obesity level (>99%).

Although there are no studies of children to determine the clinical significance of differences in QOL scores, reports from adult populations have identified differences of 0.03 as being clinically significant and differences of as little as 0.01 as being meaningful [30–33]. By this measure, severely obese children in the current study had clinically and meaningful differences in all from the three instruments (range −0.03 to −0.09). 

There is only one other study in the USA that used the HUI3 to compare scores between normal weight and overweight/obese children [34]. This study used a convenience sample of 76 predominantly African American and Hispanic children, age of 5–18, drawn from hospital clinics. The overall HUI3 score for the entire sample was 0.79 (0.17) which is close to the HUI3 score in our population (0.80 (0.24)). Also similar to the HEALTHY study, their study did not show significant differences in HUI3 scores between the normal weight and overweight/obese groups (0.81 versus 0.78, resp.). The HEALTHY study extends these findings into a larger group of minority children and a wider range of BMI%. 

The significantly lower QOL scores in the HEALTHY study were due, in part, to lower levels of functioning reported by children in the mobility domain (bend, lift, jump, walk, and run) for the HUI2 and the speech domain (being able to be understood when speaking and being able to speak at all) for the HUI3. The low mobility score in obese and severely obese children is well documented in the literature [5, 7, 35]. 

The second domain affected among obese, but not severely obese children, was speech. An extensive review of the literature was conducted, and no other study was found showing this relationship. HUI3 was also analyzed in our study by English and Spanish responders, and there was no difference in the speech domain between groups. Because we have no explanation for this finding, further studies are needed to fully understand this association. 

After adjusting for covariates, being older, male, Hispanic, African American, and advanced Tanner stage were associated with lower QOL scores. Blood glucose and insulin, on the other hand, were not. The rate of severe obesity for children 10 or younger, 11, and 12 years of age was 5.5%, 6.2%, and 8.9%, respectively; for males and females, it was 7.7% and 6.2%, respectively; and for Hispanics, African Americans, non-Hispanic white, it was 7.3%, 8.0%, and 4.9%, respectively. QOL scores were lower in older, male, and minority children because of their higher severe obesity rates. For Tanner stage, longitudinal studies have shown that obese children have more advanced Tanner stage than their lean counterparts [36–38]. 

The strength of this study is in the use of preference-weighted QOL instruments in a large school-based cohort of ethnically diverse children. This study is unique because it involves minority children who have the highest rates of obesity, and it is important to understand the role that BMI% categories may have on these children's physical and mental function. There are only two community-based studies involving small number of minority children and none used preference-weighted QOL instruments; and the only study to use a preference-weighted QOL instrument included a small number of minority children who were enrolled in hospital clinics. 

Despite these strengths, there were three limitations we must note. First, there was a low response rate (57.0%). When we analyzed the BMI, age, ethnicity, and sex between consented and nonconsented children, however, we found no significant differences [14]. Drawing three tubes of blood to measure lipids, insulin, and glucose may have dampened response rates, but in return we collected valuable biochemistries to include as covariates. Second, children in the current study are not representative of US school children. The present study had 73% African American and Hispanic children, whereas nationally 39% of children enrolled in public schools are African American and Hispanic [39]. Nonetheless, minority and disadvantaged children were oversampled because of their higher risk for obesity and type 2 diabetes. 

Third, the algorithms for estimating HUI preference-weighted scores were not derived from children or U.S. populations. They were derived from white middle-class Canadian adults [13, 15, 16]. Health care cost and preference-weighted scores used for CEA are usually considered from a societal perspective. It is the society that usually pays health care bills, and as the budget holder, it insists on economic evaluations to inform decisions of resource allocations. To develop HUI preference scores for children, adults were asked to take risk on their children's health outcomes given several fictitious health states. They were asked, for example, if their child had a physical or mental disability, would they prefer a treatment that would decrease the child's lifespan to give him/her a better quality of life or leave the disability unchanged to preserve the longer lifespan. It is likely that parents anywhere would make decisions on what is best for their child given a medical condition similar to those made by the middle-class Canadian adults who were involved in developing the HUI preference-weighted scores. Nonetheless, preference-weighted QOL measure in children is still an incomplete science, and more research is needed to determine their discriminative and evaluative roles.

In conclusion, we found that severely obese children of ethnically diverse backgrounds had significantly lower preference-weighted QOL scores than did normal weight children in all three instruments. Being overweight and obese was related to lower preference scores in one of the three instruments. The specific domains affected were mobility and speech. Lastly, although this is the first study to evaluate the relationship between preference-weighted scores and BMI% categories in a large cohort of mostly minority children, more research is needed to validate preference-weighted QOL instruments in children. 

## Figures and Tables

**Table 1:** Clinical and demographic characteristics of HEALTHY study participants.

	*N*	%
Age		
10 or younger	91	1.8
11	3535	71.0
12	1353	27.2
Male	2336	46.9
BMI percentile		
<85	2456	49.3
85–94	1003	20.1
95–98	1176	23.6
99+	344	6.9
Fasting glucose (mg/dL)		
<100	4172	83.8
100–110	745	15.0
110+	62	1.2
Fasting insulin ≥ 30 (*μ*U/mL)	326	6.5
Tanner stage		
1	492	10.0
2	1280	26.1
3	1964	40.0
4 or 5	1170	23.9
Race/ethnicity		
Hispanic	2857	57.4
Black	1066	21.4
White	1056	21.2
Family history of diabetes	649	17.8
Highest educational grade attained in household		
No HS diploma	1308	27.0
Some college	2620	54.1
College degree or higher	912	18.8

	Mean	SD

Height (cm)	150.9	7.6
Weight (kg)	51.6	15.2
BMI	22.4	5.4
Fasting glucose (mg/dL)	93.5	6.6
Fasting insulin (*μ*U/mL)	13.3	11.4

**Table 2:** Unadjusted preference-weighted QOL scores stratified by clinical and demographic characteristics.

	*N*	Health Utility Index 2		Health Utility Index 3		Feeling thermometer	
		Mean	(SD)	Mean	(SD)	Mean	(SD)
BMI%							
<85	2456	0.853	(0.157)	0.805	(0.233)	0.826	(0.156)
85–94	1003	0.848	(0.157)	0.795	(0.236)	0.803	(0.155)
95–99	1176	0.838	(0.163)	0.786	(0.242)	0.784	(0.159)
99+	344	0.814	(0.175)	0.759	(0.245)	0.740	(0.189)
		<0.001		0.004		<0.001	
Fasting glucose (mg/dL)							
<100	4172	0.845	(0.160)	0.794	(0.238)	0.807	(0.161)
100–110	745	0.852	(0.160)	0.803	(0.230)	0.798	(0.161)
100+	62	0.815	(0.173)	0.779	(0.262)	0.790	(0.179)
		0.135		0.438		0.346	
Fasting insulin (*μ*U/mL)							
<30	4653	0.847	(0.159)	0.797	(0.237)	0.809	(0.159)
30+	326	0.830	(0.168)	0.781	(0.237)	0.761	(0.181)
		0.088		0.272		<0.001	
Age							
10 or younger	91	0.849	(0.174)	0.790	(0.250)	0.808	(0.187)
11	3535	0.852	(0.153)	0.806	(0.225)	0.810	(0.158)
12	1353	0.829	(0.174)	0.768	(0.262)	0.795	(0.166)
		<0.001		<0.001		0.112	
Sex							
Male	2336	0.847	(0.160)	0.799	(0.238)	0.811	(0.154)
Female	2643	0.845	(0.160)	0.793	(0.236)	0.800	(0.167)
		0.697		0.331		0.006	
Tanner stage							
1	492	0.872	(0.147)	0.821	(0.220)	0.802	(0.162)
2	1280	0.851	(0.149)	0.803	(0.221)	0.811	(0.155)
3	1964	0.845	(0.161)	0.798	(0.238)	0.808	(0.160)
4 or 5	1170	0.830	(0.174)	0.773	(0.256)	0.798	(0.168)
		<0.001		<0.001		0.150	
Family history of diabetes							
Yes	649	0.832	(0.168)	0.773	(0.256)	0.786	(0.167)
No	3004	0.850	(0.157)	0.802	(0.232)	0.810	(0.159)
		0.014		0.007		0.002	
Race/ethnicity							
Hispanic	2857	0.836	(0.163)	0.779	(0.244)	0.791	(0.164)
Black	1066	0.849	(0.159)	0.802	(0.235)	0.825	(0.159)
White	1056	0.870	(0.149)	0.834	(0.214)	0.826	(0.152)
		<0.001		<0.001		0.019	
Highest educational grade attained in household							
No HS diploma	1308	0.833	(0.167)	0.773	(0.251)	0.789	(0.166)
Some college	2620	0.849	(0.161)	0.799	(0.234)	0.807	(0.162)
College grade or higher	912	0.859	(0.144)	0.825	(0.214)	0.827	(0.153)
		0.015		<0.001		0.009	

**Table 3:** Adjusted* differences of preference-weighted QOL scores by clinical and demographic characteristics compared with reference categories**.

	Health Utility Index 2		Health Utility Index 3		Feeling thermometer	
	Difference	(*P*-value)	Difference	(*P*-value)	Difference	(*P*-value)
BMI%						
85–94	−0.007	(0.330)	−0.018	(0.084)	−0.025	(<0.001)
95–99	−0.009	(0.178)	−0.015	(0.128)	−0.040	(<0.001)
99+	−0.030	(0.013)	−0.039	(0.025)	−0.087	(<0.001)
	(0.078)		(0.068)		(<0.001)	
Fasting glucose (mg/dL)						
100–110	0.005	(0.551)	0.010	(0.396)	0.003	(0.697)
100+	−0.030	(0.205)	−0.026	(0.475)	−0.019	(0.431)
	(0.357)		(0.520)		(0.666)	
Fasting insulin (*μ*U/mL)						
30+	0.004	(0.720)	0.014	(0.426)	−0.009	(0.419)
Age						
10 or younger	0.002	(0.909)	−0.006	(0.824)	−0.030	(0.121)
12	−0.022	(<0.001)	−0.032	(<0.001)	−0.013	(0.041)
	(0.003)		(0.003)		(0.044)	
Sex						
Male	0.000	(0.992)	0.006	(0.491)	0.014	(0.023)
Tanner stage						
2	−0.027	(0.006)	−0.023	(0.124)	0.013	(0.200)
3	−0.032	(<0.001)	0.024	(0.088)	0.006	(0.552)
4 or 5	−0.042	(<0.001)	−0.039	(0.017)	0.014	(0.190)
	(0.002)		(0.128)		(0.381)	
Family history of diabetes						
Yes	−0.011	(0.109)	−0.019	(0.065)	−0.011	(0.104)
	(0.109)		(0.065)		(0.104)	
Race/ethnicity						
Hispanic	−0.029	(<0.001)	−0.041	(<0.001)	−0.008	(0.213)
Black	−0.020	(0.017)	−0.034	(0.006)	0.005	(0.533)
	(<0.001)		(<0.001)		(0.156)	
Highest educational grade attained in household						
No HS diploma	−0.006	(0.524)	−0.020	(0.127)	−0.008	(0.383)
Some college	0.004	(0.588)	−0.006	(0.623)	−0.006	(0.421)
	(0.368)		(0.250)		(0.651)	

*Linear mixed models adjusted for age, sex, blood glucose and insulin, Tanner stage, race/ethnicity, family history of diabetes, and educational attainment.

**Reference groups were normal weight, fasting glucose < 100 mg/dL, fasting insulin < 30 *μ*U/mL, age of 11 years, female, Tanner stage 1, no family history of diabetes, non-Hispanic white children, and college graduate.
